# Correction: Simultaneous quantification of five biomarkers in ethanolic extract of *Cassia occidentalis* Linn. stem using liquid chromatography tandem mass spectrometry: application to its pharmacokinetic studies

**DOI:** 10.1039/d1ra90162a

**Published:** 2021-10-25

**Authors:** Mohammed Riyazuddin, Athar Husain, Saurabh Verma, Roshan Katekar, Richa Garg, Sudhir Kumar, Sabbu Satish, Rakesh Maurya, Tadigoppula Narender, Naibedya Chattopadhyay, Jiaur R. Gayen

**Affiliations:** Pharmaceutics & Pharmacokinetics Division, CSIR-Central Drug Research Institute Sector-10, Jankipuram Extension, Sitapur Road Lucknow-226031 India jr.gayen@cdri.res.in +91 2772450 ext. 4845; Medicinal & Process Chemistry Division, CSIR-Central Drug Research Institute Lucknow-226031 India; Endocrinology Division, CSIR-Central Drug Research Institute Lucknow-226031 India; Academy of Scientific and Innovative Research (AcSIR) Ghaziabad 201002 India

## Abstract

Correction for ‘Simultaneous quantification of five biomarkers in ethanolic extract of *Cassia occidentalis* Linn. stem using liquid chromatography tandem mass spectrometry: application to its pharmacokinetic studies’ by Mohammed Riyazuddin *et al.*, *RSC Adv.*, 2020, **10**, 4579–4588. DOI: 10.1039/C9RA07482A

The authors regret that the one of the affiliations (affiliation d) was incorrectly shown in the original manuscript. The corrected list of affiliations is as shown above.

The Royal Society of Chemistry apologises for these errors and any consequent inconvenience to authors and readers.

## Supplementary Material

